# A case of severe hypothyroidism due to lenalidomide

**DOI:** 10.1002/ccr3.2362

**Published:** 2019-08-08

**Authors:** Rachel A. Blair, Rebecca Karp Leaf, David E. Leaf

**Affiliations:** ^1^ Division of Endocrinology, Diabetes, and Hypertension Brigham and Women's Hospital Boston Massachusetts; ^2^ Division of Hematology Massachusetts General Hospital Boston Massachusetts; ^3^ Division of Renal Medicine Brigham and Women's Hospital Boston Massachusetts

**Keywords:** endocrinology, hypothyroidism, lenalidomide, multiple myeloma (Neoplasia‐ myeloma and other plasma cell dyscrasias), rhabdomyolysis

## Abstract

Lenalidomide, an immunomodulatory drug often used to treat multiple myeloma, can cause hypo‐ or hyperthyroidism. We present a patient being treated with lenalidomide for 2 years who developed severe hypothyroidism that was complicated by rhabdomyolysis and acute kidney injury. Thyroid function tests should be serially monitored in patients taking lenalidomide.

## 1 INTRODUCTION

Lenalidomide, a derivative of thalidomide, is an immunomodulatory drug commonly used for the treatment of multiple myeloma (MM).^1^ Although lenalidomide's efficacy in this setting has been well established and is generally well tolerated, lenalidomide can also cause a variety of adverse effects, including myelosuppression, venous thromboembolism, peripheral neuropathy, and thyroid dysfunction.^1^, ^2^, ^3^ Thyroid abnormalities, including both hypo‐ and hyperthyroidism, occur in approximately 5%‐10% of patients treated with lenalidomide, and most reported cases are mild.^2^, ^4^ Here, we present a case of severe hypothyroidism due to lenalidomide, resulting in multi‐organ failure including rhabdomyolysis and acute kidney injury (AKI).

## 2 CASE HISTORY

A 49‐year‐old man with MM presented to an outpatient hematology clinic with mild muscle cramps, 5‐kg weight gain, dry skin, and swelling around the eyes. Two years earlier, he was diagnosed with IgG kappa MM and was treated with three cycles of lenalidomide, bortezemib, and dexamethasone. He then received a consolidative autologous hematopoietic stem cell transplant and was initiated on lenalidomide maintenance therapy at a dose of 10 mg daily. He was maintained on this dose of lenalidomide for 2 years. The patient also had a history of two provoked pulmonary emboli, depression, and gastroesophogeal reflux disease. In addition to lenalidomide, his medications included warfarin, sertraline, omeprazole, and a multivitamin. There was a family history of thyroid disease in a maternal aunt.

On examination, the patient appeared fatigued and was noted to have periorbital edema and 1+ reflexes. His laboratory studies revealed a serum creatinine (SCr) of 1.8 mg/dL (13.9 mmol/L), which had increased from a baseline of 1.0 mg/dL (8.8 mmol/L) 4 months prior. His hemoglobin was 12.9 g/dL (reference range, 13.5‐18.0 g/dL), which had been stable over the last year. Urinalysis demonstrated 1+ blood, whereas the urine sediment was notable for absence of red blood cells (RBCs). Due to concern for myoglobinuria, a serum creatinine kinase (CK) level was measured and was found to be 11 041 U/L (reference range, 39‐308 U/L). He was admitted to the hospital for additional evaluation.

Upon further questioning, the patient denied any extreme physical activity, illicit drug use, or ingestion of myotoxic medications such as statins. A thyroid‐stimulating hormone (TSH) level was markedly elevated at 104.4 µU/L (reference range, 0.5‐5.7 µU/L). Additional thyroid function tests (TFTs) and other laboratory studies are shown in Table 1. The patient was given 6 L of intravenous normal saline, and his SCr and CK began to downtrend within 1 day. He was initiated on oral levothyroxine at a weight‐based dose of 1.6 mcg/kg, and within 3 weeks, his fatigue, weight gain, and periorbital edema had improved; his SCr also subsequently decreased to 1.2 mg/dL (9.4 mmol/L; Table 1). Two months following his initial presentation, the patient's TSH had nearly normalized to 12.66 µU/L. His levothyroxine dose was increased, and his TSH fully normalized to 4.61 µU/L within 4 months.

**Table 1:** Laboratory data on presentation and at 2‐week follow‐up

Blood tests	Reference range	On presentation	2 wk later
Thyroid function tests			
Thyroid stimulating hormone, µU/L	0.5‐5.7	104.4	69.94
Free T4, ng/dL	0.9‐1.7	<0.2	0.7
Total T3, ng/dL	80‐200	<20	–
Antithyroid peroxidase antibodies, IU/mL	0‐33.9	<10	–
Serum chemistries			
Sodium, mmol/L	135‐145	140	142
Potassium, mmol/L	3.5‐5	4.0	4.4
Chloride, mmol/L	98‐108	101	104
Bicarbonate, mmol/L	23‐32	25	28
Blood urea nitrogen, mmol/L	3.2‐8.9	4.6	5.7
Creatinine, mmol/L	3.8‐9.2	13.9	9.4
Creatinine kinase, U/L	39‐308	11,041	1,569
Lactate dehydrogenase, U/L	107‐231	498	192

### 2.1 Differential diagnosis

Regarding the etiology of the patient's hypothyroidism, his TFTs had been checked 18 months earlier and were within the normal range. Testing for antithyroid peroxidase antibodies was negative, indicating that the hypothyroidism was unlikely to be autoimmune in nature. Given normal prior TFTs, negative testing for antithyroid peroxidase antibodies, and no other clear etiology of hypothyroidism, this patient's thyroid abnormalities were attributed to lenalidomide.

In a patient with positive “blood” on urine dipstick testing—which detects heme rather than intact RBCs—but with absence of RBCs visible on the urine sediment, the differential diagnosis includes myoglobinuria due to rhabdomyolysis and hemoglobinuria due to intravascular hemolysis. Because of the urinalysis findings in this patient, a CK level was ordered, and the marked CK elevation confirmed the diagnosis of rhabdomyolysis.

The differential diagnosis for rhabdomyolysis is wide and includes physical causes such as trauma, exertion, and fluctuations in body temperature, as well as nonphysical etiologies such as infections, drugs or medications, and electrolyte disorders.^5^ Rarely, endocrinopathies, including hypothyroidism, can lead to rhabdomyolysis.^5^, ^6^, ^7^ Many patients with hypothyroidism have mild elevations in CK, but they seldomly develop clinical evidence of rhabdomyolysis, particularly in the absence of other precipitating factors such as vigorous exercise or concurrent use of statins.^6^, ^7^ The mechanisms of hypothyroidism‐induced CK elevations and rhabdomyolysis are not fully understood, but impaired glycogenolysis and mitochondrial oxidative dysfunction may both play a role.^6^, ^8^ In a study of patients with subclinical hypothyroidism (with normal TSH but low free thyroid hormone levels), patients developed higher lactate levels with exercise compared with control patients.^8^ The elevated lactate in patients with subclinical hypothyroidism is evidence of dysfunction in mitochondrial oxidative metabolism. In another study, patients with hypothyroidism were found to have atrophy of type II muscle fibers and a low rate of ATP turnover, again illustrating that skeletal muscle is a target organ for thyroid hormone and affected by thyroid hormone levels in a variety of ways.^9^

## 3 DISCUSSION

Lenalidomide is an immunomodulatory drug with antiproliferative and antiangiogenic properties, approved for the treatment of a variety of hematologic disorders.^1^ Similar to other drugs in this class, thyroid dysfunction is a known side effect of lenalidomide. The exact mechanisms by which lenalidomide causes hypothyroidism are unknown, but theories include inhibition of hormone secretion, a reduction of iodine uptake by the thyroid, as well as ischemic or autoimmune effects.^4^

The severity of hypothyroidism in the present case, however, is striking. In the largest published series to date, 10 out of 152 patients (7%) who did not have a preexisting thyroid abnormality developed hypothyroidism (n = 6) or hyperthyroidism (n = 4) following treatment with lenalidomide.^2^ Among those who developed hypothyroidism, the highest TSH concentration observed was 26.5 µU/L; the other five patients all had values that ranged from 5.64 to 8.00 µU/L. In comparison, our patient's TSH was 104.4 µU/L. Importantly, clinical symptoms of thyroid dysfunction, such as fatigue and constipation, overlap with side effects of lenalidomide, making a diagnosis of hypothyroidism challenging in these patients.^2^, ^4^ Therefore, measurement of the TSH level before lenalidomide initiation and every 2‐3 months while on treatment has been suggested.^4^

In conclusion, we report a case of lenalidomide‐induced severe hypothyroidism resulting in rhabdomyolysis and AKI in a patient with MM. As lenalidomide causes hypothyroidism in 5%‐10% of patients and symptoms are often difficult to distinguish from medication side effects, thyroid function tests should be serially monitored in these patients.

## CONFLICT OF INTEREST

None declared.

## AUTHOR CONTRIBUTIONS

Rachel Blair, MD: Treated the patient, completed a literature review, wrote and edited the paper. Rebecca K. Leaf: Identified additional hematology references. Wrote and edited the paper. David E. Leaf: Treated the patient, wrote and edited the paper.
